# Extensive ssDNA end formation at DNA double-strand breaks in non-homologous end-joining deficient cells during the S phase

**DOI:** 10.1186/1471-2199-8-97

**Published:** 2007-10-26

**Authors:** Karin H Karlsson, Bo Stenerlöw

**Affiliations:** 1 Division of Biomedical Radiation Sciences, Department of Oncology, Radiology and Clinical Immunology, Rudbeck Laboratory, Uppsala University, SE-751 85 Uppsala, Sweden

## Abstract

**Background:**

Efficient and correct repair of DNA damage, especially DNA double-strand breaks, is critical for cellular survival. Defects in the DNA repair may lead to cell death or genomic instability and development of cancer. Non-homologous end-joining (NHEJ) is the major repair pathway for DNA double-strand breaks in mammalian cells. The ability of other repair pathways, such as homologous recombination, to compensate for loss of NHEJ and the ways in which contributions of different pathways are regulated are far from fully understood.

**Results:**

In this report we demonstrate that long single-stranded DNA (ssDNA) ends are formed at radiation-induced DNA double-strand breaks in NHEJ deficient cells. At repair times ≥ 1 h, processing of unrejoined DNA double-strand breaks generated extensive ssDNA at the DNA ends in cells lacking the NHEJ protein complexes DNA-dependent protein kinase (DNA-PK) or DNA Ligase IV/XRCC4. The ssDNA formation was cell cycle dependent, since no ssDNA ends were observed in G_1_-synchronized NHEJ deficient cells. Furthermore, in wild type cells irradiated in the presence of DNA-PKcs (catalytic subunit of DNA-PK) inhibitors, or in DNA-PKcs deficient cells complemented with DNA-PKcs mutated in six autophosphorylation sites (ABCDE), no ssDNA was formed. The ssDNA generation also greatly influences DNA double-strand break quantification by pulsed-field gel electrophoresis, resulting in overestimation of the DNA double-strand break repair capability in NHEJ deficient cells when standard protocols for preparing naked DNA (i. e., lysis at 50°C) are used.

**Conclusion:**

We provide evidence that DNA Ligase IV/XRCC4 recruitment by DNA-PK to DNA double-strand breaks prevents the formation of long ssDNA ends at double-strand breaks during the S phase, indicating that NHEJ components may downregulate an alternative repair process where ssDNA ends are required.

## Background

The DNA double-strand break (DSB) is the most critical form of DNA damage and unrepaired or misrepaired DSBs may lead to cell death or changes in the genome stability. DSBs can be induced by ionizing radiation or cytotoxic drugs, but can also be induced endogenously when a replication fork encounters a single-strand break or by radicals formed during metabolism. Thus, in order to survive, the cell has to efficiently and correctly repair these breaks. An early step in the repair process is the recognition of DSBs by the binding of repair proteins to the DSB, which protects the ends from extensive degradation and keeps them in proximity to each other, preventing the joining of incorrect ends. Further, the proteins at the DSB ends recruit other proteins that are necessary for the repair of the break. In mammalian cells, non-homologous end-joining (NHEJ) is the major DSB repair pathway, whereby two ends are joined together, sometimes after limited end processing to make the ends ligatable [reviewed in ref. [1,2]]. The DNA dependent protein kinase (DNA-PK) plays a crucial role in this pathway; its heterodimer, Ku70/Ku80, recognizes and binds ends of double-stranded DNA (dsDNA) and then recruits the catalytic subunit of DNA-PK (DNA-PKcs or PRKDC) to the ends. The association of two DNA-PKcs molecules, one at each end, brings the broken ends together. The kinase activity of DNA-PKcs becomes activated upon DNA binding and association with another DNA-bound DNA-PK complex [3]. Autophosphorylation of DNA-PKcs is required for DSB rejoining by NHEJ and probably results in a conformational change in DNA-PKcs, enabling end modifying enzymes to gain access to the ends, and eventually in complete dissociation of DNA-PK from the DNA [4-7]. DNA Ligase IV/XRCC4 and the recently discovered XLF component [8,9] form the other protein complex belonging to the core proteins of NHEJ. This complex is responsible for joining of DSB ends and is recruited to the DSB by DNA-PK [10,11]. An alternative DSB repair mechanism in mammalian cells is homologous recombination (HR). In HR the homologous sequence on the sister chromatid is used as a template and HR is therefore a more accurate repair process than NHEJ. HR is initiated by generation of a 3'- single-stranded DNA (ssDNA) overhang at the DSB end, after which RAD51 forms nucleoprotein filaments on the ssDNA and mediates homologous pairing of DNA strands and strand exchange reactions between ssDNA and homologous dsDNA [reviewed in ref. [12,13]]. Numerous other proteins are also involved in this process. Various studies indicate that NHEJ and HR have an overlapping role [14-17]. However, HR is downregulated in the G_0_/G_1 _cell cycle phase as a result of low expression of CDK1, preventing ssDNA formation at the breaks [18,19], and thus cannot completely compensate for loss of NHEJ. Results from plasmid rejoining assays have shown that microhomology-directed joining could be independent of DNA-PK and Ligase IV/XRCC4 [20,21], indicating that alternative repair mechanisms exist besides HR in the absence of a functional NHEJ. This further supports the hypothesis that other pathways may compensate for defective NHEJ repair, a number of studies have shown that NHEJ mutant cells retain a significant fraction of fast repair [e.g. [22,23]]. In addition, it was demonstrated that even if the overall repair was slower in DNA-PK mutant cells, almost all DSBs were rejoined within 25 hours [24,25], suggesting that DNA-PKcs or Ku80 is not critical for DSB repair. In strong contrast to these observations, we recently found that there was almost total absence of fast rejoining in cells lacking either DNA-PKcs or Ku80 when artifacts in the DSB assay were eliminated [26]. Thus, it remains unclear how DSB repair pathways interact, whether loss of one pathway can be compensated by another, and what regulates the preference for a certain pathway.

Here we report on a unique attribute of NHEJ deficient cells exposed to DNA damaging agents. At repair times ≥ 1 h, processing of DSBs generates extensive ssDNA at the unrejoined ends in cells lacking DNA-PK or DNA Ligase IV/XRCC4. The ssDNA formation is cell cycle dependent, since no ssDNA ends were observed in G_1_-synchronized NHEJ deficient cells. Furthermore, the ssDNA generation has a great impact on DSB quantification by pulsed-field gel electrophoresis (PFGE), leading to an underestimation of the number of unrejoined DSBs in NHEJ deficient cells. These data suggest that in the absence of NHEJ proteins an S-phase specific process can access the DSB ends and presumably generate long ssDNA ends in an attempt to repair the breaks.

## Results

### DNA fragment detection in irradiated NHEJ deficient cells is dependent on the DNA extraction temperature

**Figure 1:**
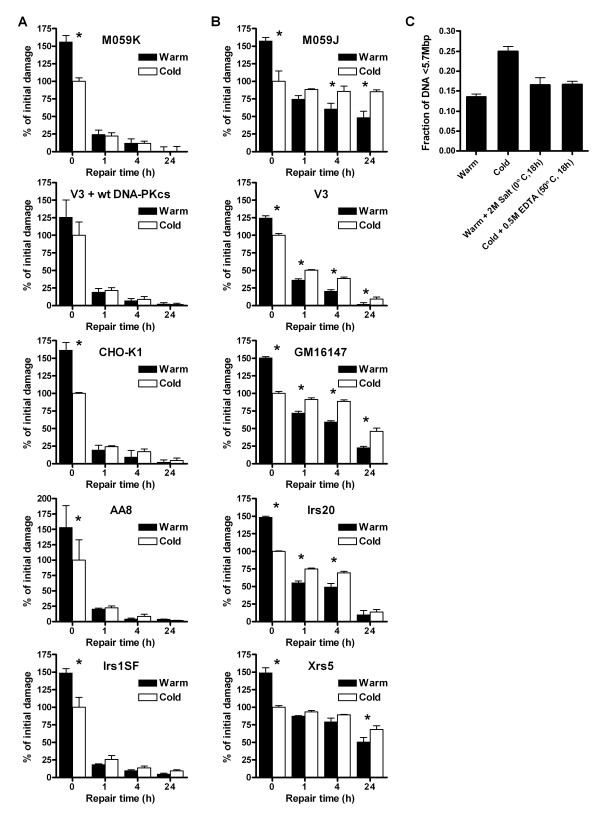
**Heat reduced mobility of unrejoined DNA fragments in NHEJ deficient cells**. (**A**) NHEJ proficient cells (M059K, V3+wt DNA-PKcs, CHO-K1, AA8 and Irs1SF (XRCC3 mutant)) or (**B**) NHEJ deficient cells (M059J (DNA-PKcs mutant), V3 (DNA-PKcs mutant), Irs20 (DNA-PKcs mutant), GM16147 (XRCC4 mutant) and Xrs5 (Ku80 mutant)) were irradiated and were allowed to repair the DNA for up to 24 h. Naked DNA was produced either by a warm or cold DNA extraction protocol and was separated by PFGE. The amount of damage detected at t = 0 h in cells lysed by the cold protocol was set to 100% (cont. on opposite page). The damage above this level detected by the warm DNA extraction protocol at 0 h consists of non-true DSBs due to the inclusion of artifactual DSBs at 50°C; these non-true DSBs were repaired within 1 h after irradiation. (**C**) 24 h after irradiation M059J (NHEJ deficient) cells were lysed with four different protocols: 1. Warm DNA extraction (50°C, 18 h); 2. Cold DNA extraction (0°C, 2 × 18 h); 3. Warm DNA extraction (50°C, 18 h) + 2 M Salt (0°C, 18 h); 4. Cold DNA extraction (0°C, 2 × 18 h) + 0.5 M EDTA (50°C, 18 h). Error bars represent the SD of at least three experiments. *Denotes significant difference with paired t-test (p < 0.05) between warm and cold extraction.

The extraction of naked genomic DNA in PFGE assays is strongly influenced by the cell lysis conditions and to avoid inclusion of artifactual DSBs in DNA preparations, we had previously developed a new cold DNA extraction protocol (Table 1 and [26]). Here we compared this new protocol with the standard warm protocol in measurements of the DSB rejoining capability of a number of repair proficient and deficient cell lines. In many of the cell lines studied there was no difference between the two protocols in the number of unrejoined DNA fragments present after ≥ 1 h of repair (Fig. 1A). However, cell lines lacking a functional NHEJ such as M059J (DNA-PKcs deficient), V3 (DNA-PKcs deficient), Irs20 (DNA-PKcs deficient), Xrs5 (Ku80 deficient) and GM16147 (XRCC4 deficient) were all exceptions: after ≥ 1 h of repair in these NHEJ deficient cells, the cold DNA extraction released up to 100% more unrejoined DNA fragments than the warm protocol (Fig. 1B). Furthermore, these cells almost completely lack fast DSB repair. Note that the higher number of DSBs detected by the warm DNA extraction protocol at the initial time point (t = 0 h) is caused by the inclusion of artifactual DSBs released by heat [26]. Clearly, warm DNA extraction overestimates the DSB rejoining capability in NHEJ deficient cells. Irradiation of DNA-PKcs deficient M059J cells within the dose-range 5–80 Gy showed similar difference between the warm and cold lysis after 20 h repair and the difference was independent of cell concentration (data not shown).

**Table 1:** DNA extraction protocols

**Name**	**Conditions**
Warm	ESP^a ^50°C 18 h
Cold	ESP 0°C 18 h + HS^b ^0°C 18 h

^a ^ESP: 0.5 M EDTA, 2% N-lauroylsarcosine, 1 mg/ml Proteinase K, pH 8.0

^b ^HS: 1.85 M NaCl, 0.15 M KCl, 5 mM MgCl_2_, 2 mM EDTA, 4 mM Tris, 0.5% TritonX-100, pH 7.5

The difference in the number of unrejoined DNA fragments detected by the two protocols in NHEJ deficient cells could be due to the difference in temperature between the two protocols (50°C and 0°C, respectively) or in the salt concentration (0 M and 2 M, respectively). In Figure 1C the number of DNA fragments extracted with four different protocols is shown for M059J cells at 24 h after irradiation. Warm, 50°C, extraction followed by 2 M salt treatment did not release more DNA fragments into the PFGE gel (protocol 3 compared to protocol 1). In contrast, cold DNA extraction followed by incubation at 50°C decreased the number of DNA fragments into the gel (protocol 4 compared to protocol 2).

Thus, heat treatment of unrejoined DNA fragments in NHEJ deficient cells reduced the DNA mobility in PFGE gels. We speculated that incubation at 50°C could result in the fusion of DNA fragments, perhaps by hybridization of ssDNA ends at DSB sites. This proposed fusion of fragments would require cellular processing of DSB ends (≥ 1 h repair) and would be unique for cells lacking NHEJ components.

### ssDNA formation at DSBs in NHEJ deficient cells

**Figure 2:**
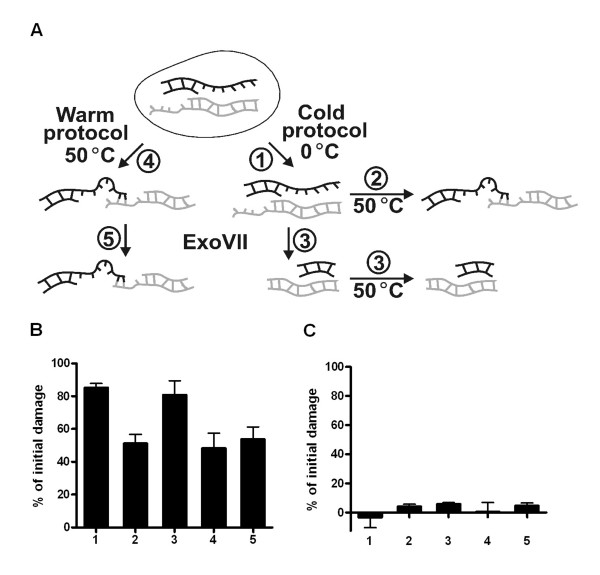
**ssDNA end formation at DSBs in NHEJ deficient cells**. Cells 24 h after irradiation were treated in five different ways (**A**): 1. Cold DNA extraction; 2. Cold DNA extraction + 18 h at 50°C; 3. Cold DNA extraction + ExoVII + 18 h at 50°C; 4. Warm DNA extraction; 5. Warm DNA extraction + ExoVII. In (**B**) and (**C**) the relative amount of initial damage in the NHEJ deficient M059J cell line and the NHEJ proficient M059K cell line, respectively are shown after the different treatments outlined in A. The level of damage at 0 h with the cold DNA extraction protocol was set to 100%. The error bars represent the SD of three experiments (M059J) or the maximum deviations of two experiments (M059K).

To test for the presence of ssDNA ends, which may lead to fusion of DNA fragments as hypothesized above, the naked DNA created from the lysis of cells at 24 h after irradiation was treated with Exonuclease VII (ExoVII), which only digests ssDNA but not dsDNA [27]. In Figure 2A five different treatments are shown. In treatment 1 the cells were lysed with the cold DNA extraction and in 2 the DNA was first extracted with the cold lysis and then the naked DNA was incubated at 50°C for 18 h. The heating of the DNA resulted in detection of almost 40% less DNA fragments in the NHEJ deficient M059J cells (compare treatments 1 and 2 in Figure 2B). However, when the DNA was incubated with ExoVII after cold DNA extraction and then incubated at 50°C, there was no decrease in the number of DNA fragments (compare treatments 3 and 1 in Figure 2B). Generation of naked DNA by the warm protocol (treatment 4) followed by ExoVII incubation (treatment 5) resulted in the same number of DNA fragments as in treatment 2. In the NHEJ proficient M059K cells none of the treatments showed any significant difference in the number of DNA fragments detected 24 h after irradiation (Fig. 2C), or 1 h after irradiation (data not shown). These results strongly suggest that ssDNA ends are created at DSBs in NHEJ deficient cells and that heat treatment of ssDNA ends reduces the amount of mobile DNA in the PFGE gel. However, since ssDNA ends should also hybridize at 0°C, the heat treatment may modify ssDNA ends in such way that they are more likely to anneal (during the following washing and electrophoresis performed at 0–10°C). Elimination of ssDNA by Exonuclease VII digestion the heating (treatment 3) prevented possible DNA hybridization and restored the DNA mobility. In treatments 2 and 4 the ssDNA ends were still available for hybridization, reducing the DNA mobility in PFGE gels.

**Figure 3:**
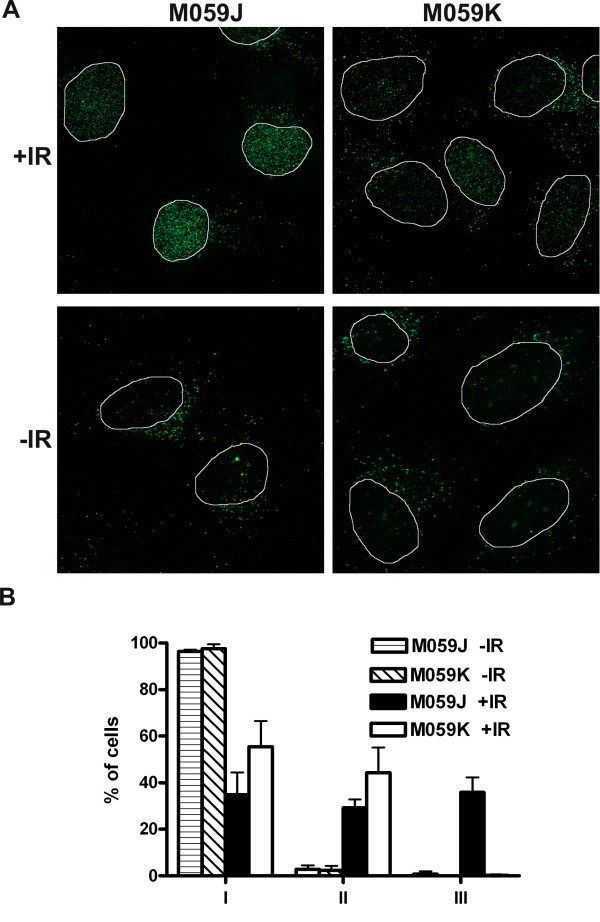
**ssDNA (BrdU foci) in NHEJ deficient cells**. M059J (NHEJ deficient) and M059K (NHEJ proficient) cells were fixed 4 h after irradiation. (**A**) BrdU corresponding to ssDNA was detected with immunofluorescence, and (**B**) cells were scored as belonging to one of the following categories: I. Weak staining in the whole nucleus; II. Increased staining in the nucleus compared to I, dots are present in the whole nucleus; III. Strong staining in the nucleus, many dots with high intensity in the whole nucleus. At least 100 cells in three experiments were scored. Error bars represent the SD.

To further test for the presence of ssDNA, the thymidine analogue BrdU was incorporated into the DNA of cells and ssDNA was visualized by using an antibody that only recognizes BrdU in ssDNA but not in dsDNA [28]. M059J and M059K cells with incorporated BrdU were irradiated and fixed after 4 h of repair. A distinct difference was noted between the cell lines M059K (NHEJ proficient) and M059J (NHEJ deficient) (Fig. 3A). About 35% of the M059J cells displayed very strong punctuate BrdU staining (type III staining) in the whole nucleus after 4 h, whereas no M059K cells displayed this staining pattern (Fig. 3B). The BrdU staining in M059J and M059K cells at 0.1 h after irradiation was similar to that in unirradiated cells and showed no type III staining (data not shown). This is additional evidence that formation of long ssDNA ends does indeed occur in NHEJ deficient cells after induction of DSBs.

### DNA Ligase IV/XRCC4 recruitment by DNA-PK to DSBs is necessary to prevent the formation of ssDNA at the ends

**Figure 4:**
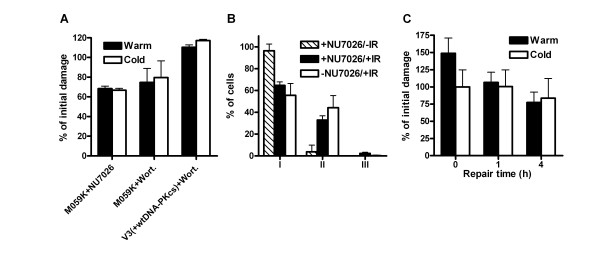
**Inhibition of DNA-PKcs autophosphorylation does not result in ssDNA formation**. (**A**) The catalytic activity of DNA-PKcs was inhibited by 50 μM NU7026 or 50 μM wortmannin in M059K cells and V3+wild type DNA-PKcs cells. At 4 h after irradiation the DNA was extracted either with the warm or the cold protocol and the number of DNA fragments was detected by PFGE. The number of DNA fragments detected at 0 h by the cold protocol was set to 100%. The error bars represent the maximum deviation of two experiments (M059K+NU7026), the standard error of the mean of three experiments (M059K+Wortmannin), and the maximum deviation of duplicates from one experiment (V3+wt DNA-PKcs). (**B**) BrdU corresponding to ssDNA was detected 4 h after irradiation in M059K cells treated with 50 μM NU7026 and cells were scored as in Figure 3. Irradiated M059K cells without NU7026 are plotted for comparison. At least 100 cells in three experiments were scored. (**C**) V3 cells expressing DNA-PKcs with six autophosphorylation mutated sites (ABCDE) were treated as in (**A**) after irradiation. Error bars represent the SD of at least three experiments.

**Figure 5:**
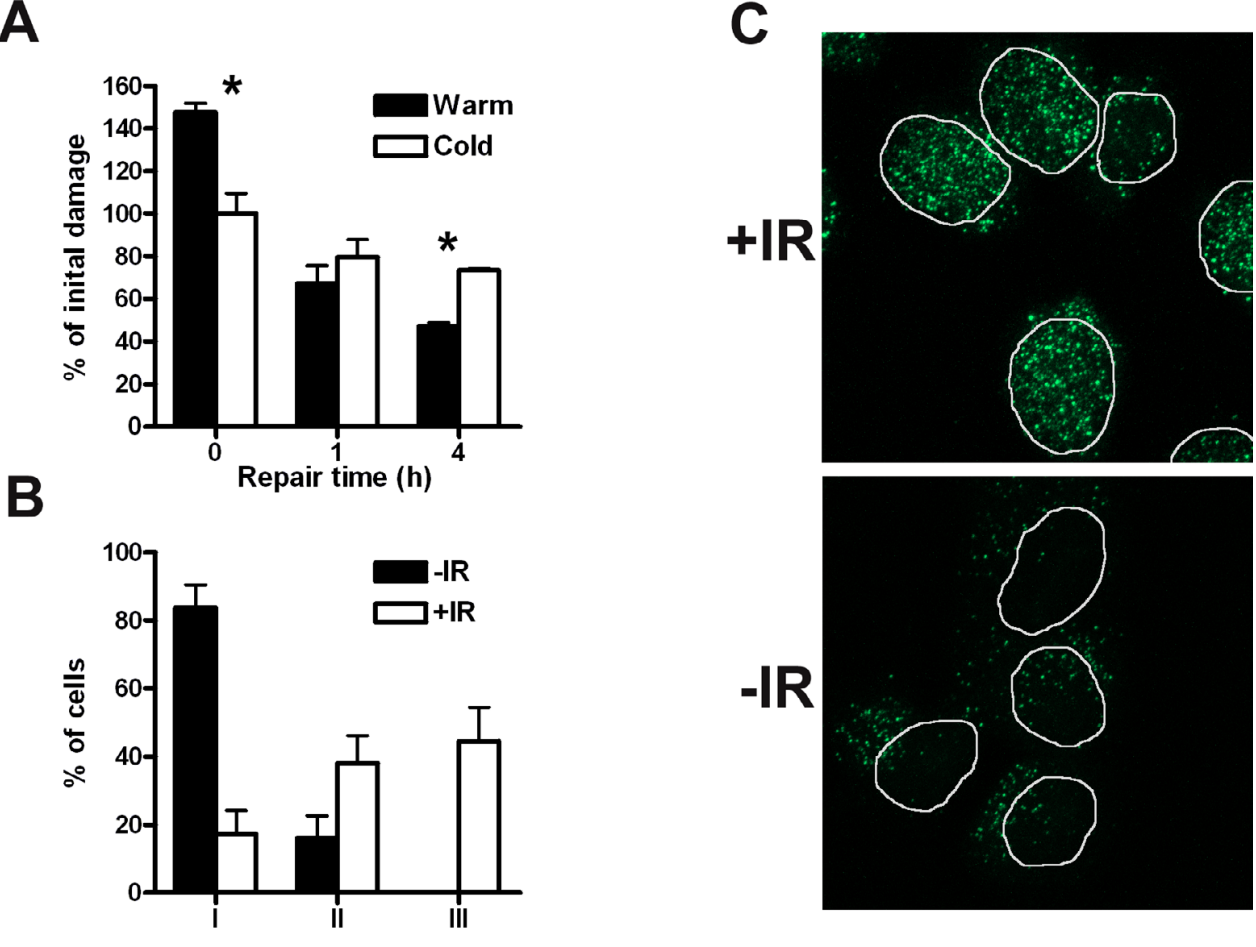
**Inhibition of the catalytic activity of DNA-PKcs in XRCC4 deficient cells does not prevent the processing of DSB ends into ssDNA**. (**A**) GM16147 (XRCC4 deficient) cells were incubated with 50 μM NU7026 and the DNA was extracted with the warm or cold protocol at different repair times after irradiation. The number of DNA fragments was determined by PFGE and normalized to the number of DNA fragments at 0 h by the cold protocol. Error bars represent the SD of three experiments. *Denotes significant difference with paired t-test (p < 0.05) between warm and cold extraction. (B and C) BrdU corresponding to ssDNA was detected 4 h after irradiation in GM16147 cells treated with 50 μM NU7026 and cells were scored as belonging to one of the following categories: I. Weak BrdU staining in the whole nucleus; II. Increased BrdU staining in the nucleus compared to I or > 20 strong dots in the nucleus; III. Strong dots in the whole nucleus and background staining in the whole nucleus. At least 100 cells in three experiments were scored. Error bars represent the SD.

To test the availability of DSB ends for single-strand processing when fast and efficient rejoining of DSBs by NHEJ is not possible, the kinase activity of DNA-PKcs was inhibited by wortmannin or NU7026. The inhibition of autophosphorylation prevents the release of DNA-PKcs from the DNA [10,29] and hence the rejoining of DSBs. In M059K cells and V3 cells complemented with wt DNA-PKcs, which were irradiated in the presence of wortmannin or NU7026, 70% or more of the DNA fragments were still unrejoined after 4 h (Fig. 4A) compared to < 10% in non-inhibited cells (Fig. 1A). However, the number of DNA fragments detected did not differ between the two DNA extraction protocols, in contrast to the findings in cells lacking DNA-PK such as M059J and V3. The lack of ssDNA formation in DNA-PKcs inhibited cells was confirmed by immunofluorescence detection of BrdU. After 4 h of repair no increase in BrdU staining, corresponding to ssDNA, was detected in M059K cells incubated with NU7026, as compared to that in cells not incubated with the DNA-PK kinase inhibitor (Fig. 4B). The formation of ssDNA appears to be dependent on the absence of DNA-PK at the DSB ends; inhibition of the kinase activity, and consequently inhibition of the rejoining of DSBs by NHEJ, is not sufficient for generation of ssDNA ends. Further evidence that DNA-PKcs protect DSB ends from resection was provided in V3 cells (DNA-PKcs deficient) transfected with a plasmid containing a mutated DNA-PKcs in six of its autophosphorylation sites (ABCDE) [6,30]. The ABCDE mutant cells have normal DNA-PKcs kinase activity but are radiosensitive, as a result of defective NHEJ, and the lack of autophosphorylation at the ABCDE sites blocks the ends from processing and ligation [6,7]. When we irradiated V3 cells expressing this ABCDE mutant form of DNA-PKcs and extracted DNA with the warm or cold protocol after different repair times, no significant difference in the number of DNA fragments detected by PFGE was seen (Fig. 4C). These results support the data showing that DNA-PKcs protects the ends from resection when present at the ends, even though it is catalytically inactive. However, when the activity of DNA-PKcs was inhibited in the XRCC4 deficient cell line GM16147, ssDNA was still formed at repair times ≥ 1 h, as detected by DNA extraction with the warm and cold protocols (Fig. 5A). In accordance with these PFGE results, the majority of the irradiated GM16147 cells with incorporated BrdU and incubated with NU7026 had a higher (II) or much higher level (III) of ssDNA 4 h after irradiation, compared to control cells (Fig. 5B and 5C). These data strongly suggest that it is the recruitment of Ligase IV/XRCC4 to the ends by DNA-PK that is the important factor for end protection; in NU7026 or wortmannin treated cells Ligase IV/XRCC4 can still bind to DSB ends, since the kinase activity of DNA-PKcs is dispensable for the binding of Ligase IV/XRCC4 to the ends [10]. Ligase IV/XRCC4 apparently prevents the generation of ssDNA ends even though ligation of the ends by NHEJ is inhibited.

### Formation of ssDNA ends at DSBs in NHEJ deficient cells in the S phase but not in the G_1 _phase of the cell cycle

**Figure 6:**
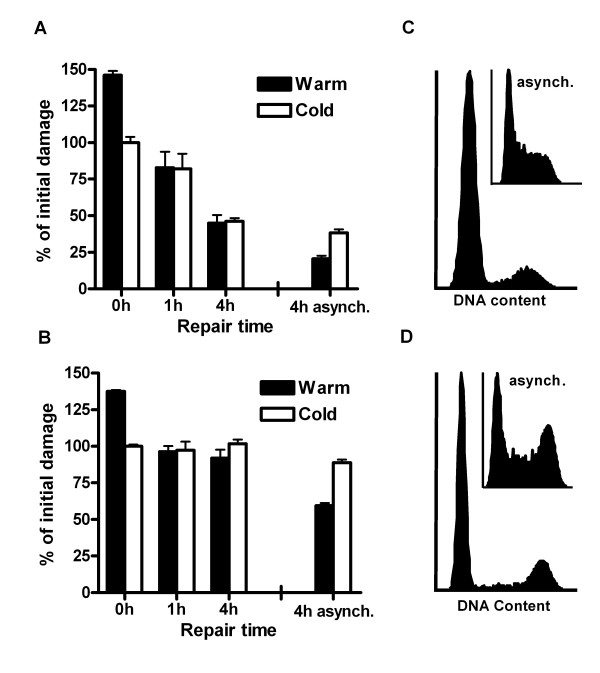
**G_1 _synchronization of NHEJ deficient cells prevents the formation of ssDNA at DSBs**. (**A**) G_1_-synchronized V3 (DNA-PKcs deficient) and (**B**) GM16147 (XRCC4 deficient) cells were irradiated and at different repair times the DNA was extracted with the warm or cold protocol. The number of DNA fragments was determined by PFGE and the number of DNA fragments detected at 0 h by the cold protocol was set to 100%. The corresponding remaining damage at 4 h in asynchronous cells is plotted for comparison. (**C**) The V3 and (**D**) GM16147 cell cycle distributions at the time of irradiation. The corresponding distributions in the asynchronous cell population are seen in the insert. Error bars represent the SD of three experiments.

To determine whether the position in the cell cycle influences the processing of DSB ends, V3 cells (deficient in DNA-PKcs) and GM16147 cells (deficient in XRCC4) were synchronized in the G_1 _phase of the cell cycle and then irradiated. In contrast to asynchronous cells, the number of DNA fragments detected in G_1 _phase cells was independent of the DNA extraction protocol used (Fig. 6A and 6B). At the time of irradiation > 85% of the synchronized V3 cells were in G_1 _(Fig. 6C), compared to 30% of the asynchronous population. In the GM16147 cells the corresponding proportions were 70% (G_1_-synchronized) and 30% (asynchronous) (Fig. 6D). The fraction of S-phase cells was < 15% in both of the synchronized cell lines. This indicates that ssDNA ends are not generated at DSBs in the G_1 _phase of the cell cycle. Additional support for the importance of cell cycle position is provided by results from DNA Ligase IV deficient human fibroblasts (GM16088), which at the time of irradiation were confluent and > 90% of the population was in G_1_; in these cells no ssDNA formation was found by the PFGE assay, since there was no difference in the number of unrejoined DNA fragments between the warm and cold DNA extraction protocols (data not shown).

**Figure 7:**
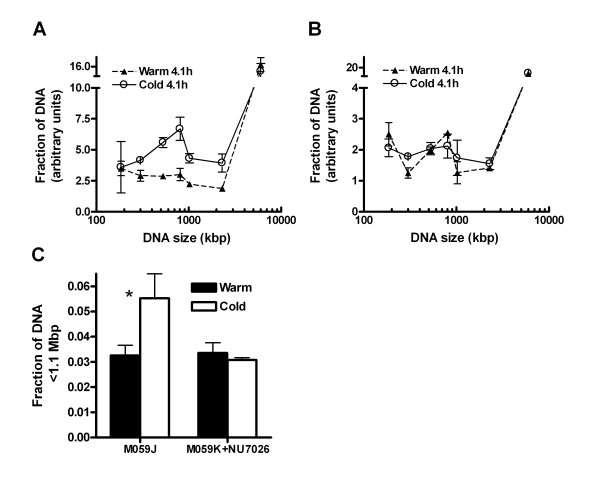
**ssDNA ends at DSBs in S-phase cells lacking DNA-PKcs**. DNA-PKcs deficient cells (M059J) and DNA-PKcs inhibited cells (M059K+NU7026) were pulsed with 3H-thymidine 1 h before irradiation with 80 Gy. The size distributions of DNA fragments containing incorporated 3H-thymidine (i.e. DNA from cells in the S phase during the 3H incubation) in M059J and M059K+NU7026 cells at 4.1 h are shown in (**A**) and (**B**), respectively. The DNA was extracted by the warm or cold protocol. The amount of fragments < 1.1 Mbp was summed and the total fraction of S-phase DNA < 1.1 Mbp, extracted by the warm and cold protocol, in M059J and M059K+NU7026 was plotted for 4.1 h (**C**). Error bars represent the SEM from three experiments (M059J) or the maximum error from two experiments (M059K+NU7026).* Denotes a significant difference with the t-test (p < 0.025).

To further reveal the possible influence of the S phase in the generation of ssDNA ends in NHEJ deficient cells, we specifically analyzed the rejoining in newly replicated DNA. Cells were labeled with a short pulse (1 h) with [^3^H]thymidine prior to irradiation. The ^3^H-labeled DNA thus only represents the newly replicated DNA, in contrast to standard uniform label with ^14^C for two cell cycles, which labels the whole genome. In Figure 7 the size distribution of DNA fragments in DNA-PKcs deficient cells (M059J) and DNA-PKcs inhibited cells (M059K + NU7026) is shown at 4 h after irradiation. M059J cells displayed a lower yield of small fragments (< 1–2 Mbp) when the DNA was extracted with the warm protocol compared to the cold protocol (Fig. 7A and 7C). In accordance with data shown in Figure 4A, no difference in DNA fragment yield between the warm and cold protocol was detected in DNA-PKcs inhibited M059K cells (Fig. 7B and 7C). These data suggest that ssDNA are generated at DSB sites in S-phase NHEJ deficient cells.

## Discussion

Here we report on extensive strand resection or unwinding of DSB ends, resulting in long ssDNA ends, in NHEJ deficient cells ≥ 1 h after DSB induction. The cause of this processing of DSB ends is not known, but one possibility is an alternative DSB repair mechanism, which attempts to rejoin the DSBs in the absence of NHEJ. Since these ssDNA ends were only detected in the S phase but not in the G_1 _phase cell cycle, HR is an obvious candidate. HR is downregulated in the G_1 _phase but active in the S and G_2 _phases and the first step in HR is the creation of ssDNA ends, which may extend as far as 1 kbp from the break [31]. CDK1 probably has an important role in this regulation and in budding yeast CDK1 is required for DSB-induced HR and efficient resection of DSB ends [18]. Several reports suggest that NHEJ and HR compete for the rejoining of DSBs; Ku proficient cells show a lower degree of HR than Ku deficient cells [32,33], supposedly as a result of the protection of ends from HR processing by Ku. This is supported by the observation that the initiation of HR is blocked by the binding of Ku to DSB ends [34]. Upregulation of HR has also been demonstrated in XRCC4 deficient cells [17,35], indicating an ability of HR to compensate for non-functional NHEJ. In addition, data from cells containing different mutations in DNA-PKcs autophosphorylation sites suggest that the phosphorylation status of DNA-PKcs may have a role in regulating the access of ends and might control the choice of pathway [36]. Interestingly, HR was blocked when the catalytic activity of DNA-PKcs was inhibited, presumably as an effect of the blocking of the end processing required for HR [37]. This is in agreement with the present finding that ssDNA formation at DSB ends was blocked in DNA-PKcs inhibited cells, although we showed that DNA Ligase IV/XRCC4 was also needed at the ends together with inhibited DNA-PKcs to prevent processing (Fig. 4 and 5). However, whether it is HR, including single strand annealing (SSA) between repetitive DNA sequences, or another process that gains access to the DSB ends in the absence of functional NHEJ and is responsible for the generation of ssDNA ends, that process is clearly not able to completely compensate for the lack of NHEJ, as a large number of unrejoined DNA fragments were still present 4–24 h after DSB induction in NHEJ deficient cells. Thus NHEJ seems to be the major repair pathway in mammalian cells throughout the cell cycle.

The ssDNA formation at unrejoined DSBs in NHEJ deficient cell lines has major implications for the quantification of DSBs by the common PFGE assay, where Mbp-DNA fragments are separated by electrophoresis. In the standard assay, naked DNA fragments are extracted from cells by incubating the cells in EDTA buffer containing a detergent and Proteinase K at 50°C. We show here that incubation at 50°C causes the ssDNA ends to fuse, resulting in fewer DNA fragments and an underestimation of the number of DSBs still present 1–24 h after DSB induction. The use of a recently developed assay [26], in which the DNA extraction is performed at 0°C prevents heat-dependent ssDNA hybridization and therefore results in a more accurate DSB quantification and in some cases a drastic decrease in the estimated rejoining capability of NHEJ deficient cell lines (Fig. 1B).

Although larger numbers of DSBs were detected with the cold protocol in all NHEJ deficient cell lines tested, the differences in the rejoining capacity observed between these cell lines with the standard protocol remained when the new cold protocol was used. The hamster cell lines V3 and Irs20, although displaying a much slower repair of DSBs compared to wild type cells, still rejoined the majority of breaks within 24 h. The same ability for rejoining was detected in asynchronous and G_1_-synchronized V3 cells (compare Figure 1B with Figure 6A), suggesting that a DNA-PKcs independent repair pathway, separated from HR and S-phase dependency, exists in the V3 cell line. In contrast to the hamster cells, no or very little rejoining of DSBs was detected up to 24 h of repair in the human glioma cell line M059J. It is possible that a mutation in ATM [38], a protein involved in DSB repair and signaling, might partly be responsible for the extreme lack of rejoining in these cells, although the low expression of ATM does not seem to influence the radiosensitivity of M059J cells [39]. In addition, hyperphosphorylation of the replication protein A2 (RPA2) in these cells, leading to decreased binding of RPA to ssDNA, could further influence the observed deficiency in DSB rejoining [40] The variation in the rejoining capability may also be due to species differences; for instance the level of DNA-PKcs is much lower in hamster cells than in human cells [37,41] or there may be residual DNA-PKcs activity in the hamster mutants [41,42]. In G_1_-synchronized XRCC4 deficient cells, no or very little rejoining was detected up to 4 h after DSB induction, indicating that no alternative end-joining independent of DNA Ligase IV/XRRC4 occurs in the G_1 _phase of the cell cycle in mammalian cells.

## Conclusion

Our data suggest that DNA binding by NHEJ proteins prevent the formation of long ssDNA ends at DSB sites during the S phase. This indicates that Ku, DNA-PKcs and XRCC4 may downregulate an alternative repair process where ssDNA ends are required. Furthermore, these ssDNA ends hybridize when standard DNA extraction protocols are used for DSB measurements by pulsed-field gel electrophoresis, causing an overestimation of the rejoining capabilities in NHEJ deficient cells. In summary, our study demonstrates the importance of DNA Ligase IV/XRCC4 and DNA-PK in both the fast repair of DSBs and the regulation of processes not directly involved in NHEJ.

## Methods

### Cell lines and chemicals

The human glioma cell lines M059K and M059J [43] (ATCC) were cultured in DMEM/Ham's F-12 supplemented with 1× non-essential amino acids. The radiosensitive Chinese Hamster Ovary cell lines Xrs5 [44] (ATCC), V3 [45], Irs1SF [46] and Irs20 [47] were cultured in Eagle's minimal essential medium (MEM). Geneticin (Sigma, St. Louis, MO), 300 μg/ml, was added to V3 cells containing a yeast artificial chromosome with wild-type DNA-PKcs [48] or a plasmid coding for mutant DNA-PKcs [30]. The Chinese Hamster Ovary cell line GM16147 [49] (Coriell) was cultured in DMEM/Ham's F-12. The Chinese Hamster Ovary cell line CHO-K1 (ATCC) was cultured in Ham's F-10. All media were supplemented with 10% fetal bovine serum (Sigma), 2 mM L-glutamine, 100 μg/ml streptomycin and 100 IU/ml penicillin. Media and supplements were from Biochrom (Berlin, Germany). The cells were grown at 37°C, 5% CO_2_. Wortmannin (Sigma), bromodeoxyuridine (BrdU; Sigma) and NU7026 (Calbiochem, Darmstadt, Germany) stocks were dissolved in DMSO.

### Irradiation of cells and quantification of DSBs

The cells were allowed to incorporate ^14^C-thymidine (1 kBq/ml) into their DNA for approximately two doubling times before irradiation. In pulse-label experiments (Fig. 7) cells were incubated with 37 kBq/ml ^3^H-thymidine for 30 min prior to irradiation. The cells were irradiated with a dose of 40 Gy unless otherwise stated. They were put on ice about 20 min before irradiation and were kept on ice during the irradiation with a ^137^Cs source (1.2 Gy/min). The irradiated cells were trypsinized (the stated repair time 0 h corresponds to 0–0.02 h; later time points differ no more than 10% from the stated time), mixed with melted agarose, and molded into plugs. They were then lysed either with warm or cold DNA extraction protocol (Table 1) and the naked DNA was separated by PFGE. The amount of DNA in different size intervals was measured by liquid scintillation and the fraction of activity released < 5.7 Mbp in the gel was used as a measure of the number of DNA fragments present. The total ^14^C activity in each lane did not vary between the two DNA extraction protocols or between irradiated and unirradiated samples, which demonstrates that these treatments did not lead to any significant degradation of DNA. To analyze rejoining of S-phase DNA in pulse-labeled cells, DNA fragment distributions below 3 Mbp were obtained as described previously [50].

### Exonuclease VII treatment

The cells were irradiated and lysed as described above and the plugs containing naked DNA were then washed 3 × 1 h in 70 mM Tris-HCl, 8 mM EDTA, with an additional wash overnight. The plugs were equilibrated in exonuclease buffer (70 mM Tris-HCl, 8 mM EDTA, 10 mM 2-mercaptoethanol, 50 μg/ml BSA) for 1 h. The plugs were kept on ice at all times. Each 20 μl plug was then incubated with 100 μl exonuclease buffer with or without 0.8 U Exonuclease VII (GE Healthcare) for 2 h at room temperature (RT). Finally, the plugs were washed 4 × 1 h in 0.5 M EDTA followed by an additional wash overnight.

### G1 synchronization and flow cytometry

8 × 10^4 ^cells/cm^2 ^were seeded and allowed to grow for 5 days in complete medium with 1–2 kBq/ml ^14^C-thymidine. The cells were then irradiated as described above and incubated with 50 μg/ml BrdU for 30 min prior to washing in PBS and fixation in ice-cold 70% ethanol. The cells were stored at 4°C for 3–7 days and the DNA was then denatured in 2 M HCl/0.5% TritonX-100 for 30 min at RT. After neutralization in 0.1 M sodium tetraborate, the cells were incubated with BrdU mouse antibody (ab) (17 μg/ml, NA61, Calbiochem) in 0.5% Tween20/1% BSA-PBS for 1 h at RT. After a wash in PBS the cells were incubated with secondary anti-mouse ab (Alexa Fluor 488, Molecular Probes, Eugene, OR) and then stained with 20 μg/ml propidium iodide + 0.1% NP40 in 100 μg/ml RNAse-PBS for at least 30 min at 4°C prior to flow cytometry analysis using a FACSort (Beckton Dickinson). The data were analyzed with the software FlowJo (Version 6.2.1, Tree Star Inc, Ashland, OR).

### Detection of ssDNA by immunofluorescence

Cells on microscope slides were grown in 10 μg/ml BrdU for two doubling times before irradiation. In some cases 50 μM NU7026 was added 1 h before irradiation. The cells were put on ice 10 min before irradiation and kept on ice during the irradiation with 40 Gy. Warm media with or without NU7026 was added and after repair of DNA for 4 h at 37°C, the cells were fixed and treated as described elsewhere [51]. Primary mouse antibody against BrdU in ssDNA was used at 4 μg/ml (NA61, Calbiochem). Images of the cells were captured with a CCD camera on a Zeiss LSM 510 Meta confocal microscope using a 40 × objective.

## Authors' contributions

KHK performed all the experiments and drafted the manuscript. BS conceived the study and participated in its design and helped to draft the manuscript. All authors read and approved the final manuscript.
